# Lymphatic Filariasis Elimination in American Samoa: Evaluation of Molecular Xenomonitoring as a Surveillance Tool in the Endgame

**DOI:** 10.1371/journal.pntd.0005108

**Published:** 2016-11-01

**Authors:** Colleen L. Lau, Kimberly Y. Won, Patrick J. Lammie, Patricia M. Graves

**Affiliations:** 1 Department of Global Health, Research School of Population Health, Australian National University, Canberra, Australia; 2 Centers for Disease Control and Prevention, Division of Parasitic Diseases and Malaria, Atlanta, United States of America; 3 Australian Institute of Tropical Health and Medicine and College of Public Health, Medical and Veterinary Sciences, James Cook University, Cairns, Australia; Wellcome Trust Sanger Institute, UNITED KINGDOM

## Abstract

The Global Programme to Eliminate Lymphatic Filariasis has made significant progress toward interrupting transmission of lymphatic filariasis (LF) through mass drug administration (MDA). Operational challenges in defining endpoints of elimination programs include the need to determine appropriate post-MDA surveillance strategies. As humans are the only reservoirs of LF parasites, one such strategy is molecular xenomonitoring (MX), the detection of filarial DNA in mosquitoes using molecular methods (PCR), to provide an indirect indicator of infected persons nearby. MX could potentially be used to evaluate program success, provide support for decisions to stop MDA, and conduct post-MDA surveillance. American Samoa has successfully completed MDA and passed WHO recommended Transmission Assessment Surveys in 2011 and 2015, but recent studies using spatial analysis of antigen (Ag) and antibody (Ab) prevalence in adults (aged ≥18 years) and entomological surveys showed evidence of possible ongoing transmission. This study evaluated MX as a surveillance tool in American Samoa by linking village-level results of published human and mosquito studies. Of 32 villages, seropositive persons for Og4C3 Ag were identified in 11 (34.4%), for Wb123 Ab in 18 (56.3%) and for Bm14 Ab in 27 (84.4%) of villages. Village-level seroprevalence ranged from 0–33%, 0–67% and 0–100% for Og4C3 Ag, Wb123 Ab and Bm14 Ab respectively. PCR-positive *Aedes polynesiensis* mosquitoes were found in 15 (47%) villages, and their presence was significantly associated with seropositive persons for Og4C3 Ag (67% vs 6%, *p*<0.001) and Wb123 Ab (87% vs 29%, *p* = 0.001), but not Bm14 Ab. In villages with persons seropositive for Og4C3 Ag and Wb123 Ab, PCR-positive *Ae*. *polynesiensis* were found in 90.9% and 72.2% respectively. In villages without seropositive persons for Og4C3 Ag or Wb123 Ab, PCR-positive *Ae*. *polynesiensis* were also absent in 94.1% and 70.6% of villages respectively. Our study provides promising evidence to support the potential usefulness of MX in post-MDA surveillance in an *Aedes* transmission area in the Pacific Islands setting.

## Author Summary

Lymphatic filariasis (LF) is caused by infection with filarial worms that are transmitted by mosquito bites. Globally, 36 million are disfigured and disabled by complications such as severe swelling of the legs (elephantiasis) or scrotum (hydrocele). The Global Programme to Eliminate LF (GPELF) aims to interrupt disease transmission through mass drug administration (MDA), and to control illness and suffering in affected persons. Significant progress has been made toward eliminating LF from many parts of the world, including the Pacific Islands. Current challenges of the GPELF include identification of any residual hotspots of ongoing transmission, and effective strategies for early identification of any resurgence of infections. As humans are the only reservoirs of LF parasites and mosquitoes have short flight ranges, one such strategy is to monitor LF infection in mosquitoes as an indicator of ongoing transmission nearby. Mosquito monitoring could potentially be used to evaluate program success, provide support for decisions to stop MDA, and conduct post-MDA surveillance. Our study evaluated mosquito monitoring as a surveillance tool in American Samoa by linking village-level results of published studies of LF in humans and mosquitoes, and provides promising evidence to support the potential usefulness of mosquito monitoring in post-MDA surveillance the Pacific Islands.

## Introduction

Lymphatic filariasis (LF) is a parasitic infection caused by *Wuchereria* or *Brugia* species of helminth worms, and transmitted by mosquito vectors including *Aedes*, *Anopheles*, *Culex* and *Mansonia* species. Globally, an estimated 68 million people are currently affected, including 36 million microfilaraemic persons and 36 million who are disabled or disfigured with complications such as severe lymphoedema, including elephantiasis and scrotal hydrocoeles [1]. The Global Programme to Eliminate LF (GPELF) aims to eliminate the disease as a public health problem by 2020 using two main strategies: i) to interrupt transmission through mass drug administration (MDA) and ii) to control morbidity and disability of affected persons. In the Pacific Islands, the Pacific Programme to Eliminate LF (PacELF) was formed in 1999 as part of GPELF to focus on 22 Pacific Island Countries and Territories (PICTs), which include >3000 islands and 8.6 million people [2].

The sustained success of elimination programs requires cost-effective assessment and monitoring after successful completion of MDA to determine whether there are any residual foci of infection, and to detect potential resurgence in a timely manner. The WHO currently recommends post-MDA surveillance using transmission assessment surveys (TAS), which use critical cut-off values of numbers of antigen-positive children aged 6–7 years to determine whether transmission has been interrupted in defined evaluation units [3]. In *Brugia* transmission areas, antibody positivity is used for TAS. Cut-off thresholds for passing TAS vary depending on population size of the target group and the local species of filarial parasites and mosquito vectors. For example, where *W*. *bancrofti* is endemic, the target cut-off value is estimated based on upper 95% confidence interval of <1% antigen prevalence if *Aedes* is the principal vector, or <2% antigen prevalence if *Anopheles* or *Culex* predominate. TAS typically involve school-based or community-based testing of 6–7 year old children. Community-based surveys are often logistically challenging, particularly in developing countries with limited financial and human resources. In some areas (e.g. most of the Pacific Islands), difficult access to dispersed populations in remote islands provides additional challenges. Also, TAS typically use rapid antigen detection tests (Filarial Immunochromatographic Test (ICT) cards or Filarial Test Strips [4]), which might have reduced sensitivity after many rounds of MDA [5,6]. TAS has been widely used to inform important programmatic decisions including stopping or restarting MDA, but recent studies suggest that in some settings (including American Samoa), TAS might not be sufficiently sensitive for determining whether transmission has been interrupted [7,8].

As elimination programs reach the endgame phases and antigen prevalence drops to very low levels, increasingly sensitive tools and strategies will be required to efficiently detect any evidence of ongoing transmission or resurgence. The WHO and GPELF have identified a number of operational challenges and unanswered questions for elimination programs, including the significance of residual microfilaraemia and antigenaemia in communities where the target threshold level has been achieved through MDA, identification of residual high-prevalence areas and strategies for managing them, and the need for development of cost-effective strategies for post-MDA surveillance [9]. As humans are the only reservoir for *W*. *bancrofti* parasites, one such strategy is to monitor mosquitoes for evidence of LF larval stages [10,11]. Dissection and microscopic examination of mosquitoes is time consuming and labour-intensive, and cannot be routinely recommended for post-MDA surveillance for practical reasons. With recent technological advances, LF molecular xenomonitoring (MX), the use of molecular methods (PCR) to detect filarial DNA in mosquitoes, has been explored and promising results are emerging [12,13]. PCR-positive mosquitoes provide an indirect indicator of the presence of infected humans and possible ongoing transmission [10,14–16]. For example, considering that the flight ranges of *Ae*. *polynesiensis* mosquitoes are on the order of a hundred metres [17], detection of PCR-positive mosquitoes in areas where these are the main vectors indicates that infected persons are or were recently nearby. Molecular methods are also more sensitive than manual dissection for detecting infections [18]. Studies have reported the ability of PCR to detect one microfilaria in pools of 50–100 mosquitoes [19], and for at least 2 weeks after mosquitoes (both vector and non-vector) ingest microfilaria-positive blood [20], which is close to the average life span of most mosquito species.

Molecular xenomonitoring has been found to be a potentially useful indicator of human LF infections with different species of mosquito vectors in diverse settings including American Samoa [11], French Polynesia [16], Egypt [12,21], Sri Lanka [8], Sierra Leone [22], and Ghana [23]. Molecular xenomonitoring is therefore potentially useful for evaluating the success of elimination programs, providing support for decisions to stop MDA, and conducting ongoing post-MDA surveillance [24]. Compared to TAS, MX has the advantages of being non-invasive to humans, and potentially more cost-effective in some settings. However, MX requires entomological expertise for trapping and processing mosquitoes, and laboratories capable of conducting large scale molecular diagnostics. In addition, there are currently unanswered questions about sampling strategies, limited evidence to inform the translation of MX results into operational strategies, and no clear guidelines on the thresholds of DNA prevalence that should be used to indicate likely ongoing transmission. Cut-off points of 0.25%, 0.5%, and 1% have been suggested for *Culex* areas [8,12,25], and 0.085% for L3 and 0.65% for any larval stage for *Anopheles* areas [13]. There are currently no clear recommendations for *Aedes* areas, but a provisional threshold of <0.1% has been suggested [26]. The lower the estimated cut-off points, the larger the sample sizes of mosquitoes that will be required for MX.

As part of the PacELF, American Samoa has made significant progress toward reducing LF infection rates. After seven rounds of MDA from 2000 to 2006, antigen prevalence in humans dropped from 16.5% (N = 3018) in the 1999 baseline assessment to 2.3% (N = 1881) in 2007 in a community cluster survey [27]. American Samoa passed TAS in 2011–2012 and again in 2015, but recently published studies using spatial analysis of antigen prevalence in adults [7] and molecular xenomonitoring [28] showed evidence of possible ongoing transmission. By linking the results of the published human and mosquito studies, we aim to evaluate MX as a surveillance tool in the post-MDA setting in American Samoa, an *Aedes* transmission area in the Pacific Islands.

## Methods

### Study location and setting

American Samoa is a US Territory in the South Pacific, consisting of a group of small tropical islands with a total population of 56,000 [29] living in 67 villages. Over 90% of the population live in small villages on the main island of Tutuila, and the remainder on the adjacent island of Aunu’u and the remote Manu’a group of islands (Ta’u, Ofu, and Olosega). *W*. *bancrofti* is the only species of human filarial worm known to be present in American Samoa. The main vector is the highly efficient day-biting *Ae*. *polynesiensis*, and other vectors include *Ae*. *samoanus* (night-biting), *Ae*. *tutuilae* (night-biting), and *Ae*. *upolensis* (day-biting) [30–32].

### Human infection data

Data were obtained from a published study on the seroprevalence and spatial epidemiology of lymphatic filariasis in American Samoa [7]. The study used samples from a serum bank collected from May to August 2010 for a leptospirosis study; the study design has been published previously [33,34]. Briefly, the study included 807 adults (aged 18 to 87 years, 52.4% males) from 659 households in 55 villages on all five inhabited islands of American Samoa. Sampling was designed to provide a representative sample of the adult population in American Samoa, in both age and geographic distribution. Using these 2010 samples, a seroprevalence study was conducted in 2013 [7], and found that 3.2% were seropositive for Og4C3 Ag, and 8.1% and 17.9% were seropositive for Wb123 Ab and Bm14 Ab, respectively [7]. The study also found significant spatial clustering of Ag-positive persons; average cluster size was 1,498m in diameter for those with Og4C3 Ag >32 units, and the proportion of the variation explained by geographic proximity was 62%. Higher infection rates were found in males and recent migrants to American Samoa. Antigen (Og4C3) positivity indicates the presence of adult worm antigen but does not provide information on the viability, e.g. the worm could be alive or dead, or there could be a single sex worm infection or sterile worm infection. The presence of Og4C3 Ag represents current or recent infection. The presence of antibodies represents current or past infection, possibly many years in the past.

For our study, human data were summarized by village and the following variables were generated:

- Total number of people sampled from each village
- Village-level seroprevalence (point estimates and 95% CI) for Og4C3 Ag, Wb123 Ab and Bm14 Ab,
- Seropositive village for Og4C3 Ag, Wb123 Ab, and Bm14 Ab (defined as villages with at least one seropositive person)

A village-level summary of the human serological data is provided in S1 Appendix.

### Molecular xenomonitoring data

Schmaedick et al conducted a MX study in American Samoa from February to June 2011, approximately 9 months after the above human serum specimens described above were collected. Detailed descriptions of the study and its findings have been published [28], and a village-level summary is provided in S1 Appendix. Briefly, mosquitoes were collected from 34 randomly selected villages on the island of Tutuila, the only village on Aunu’u, all five villages on the Manu’a Islands, and the village of Ili’ili (on Tutuila) where two ICT-positive children were identified during the 2011 TAS. Up to 10 traps were placed in each village for 24 to 48 hours, and mosquitoes were removed from traps twice daily. The study collected a total of 22,014 female mosquitoes of *Aedes* and *Culex* genera that were sorted into 2,629 pools of ≤20 mosquitoes (range 1 to 20) for PCR testing. Real-time PCR was conducted using primers designed to amplify a fragment of *W*. *bancrofti* [35]. A positive PCR result indicates the presence of filarial worm DNA, but does not provide any information on whether the worms are alive or transmissible. Each pool included only one mosquito species, except for the *Ae*. *(Finlaya)* group of species (*Ae*. *oceanicus*, *Ae*. *samoanus*, and *Ae*. *tutuilae*) which were combined for PCR testing because of morphological similarities. The MX study calculated maximum likelihood point estimates of the prevalence of PCR-positive *Ae*. *polynesiensis* for each village or village group using PoolScreen software (version 2.0.3), which takes into account the average number of mosquitoes per pool and the proportion of pools that were PCR-positive. Point estimates of village-level prevalence of PCR-positive *Ae*. *polynesiensis* ranged from 0% to 2.8% for villages on Tutuila and Aunu’u, and was 0% for all villages in the Manu’a islands. The findings indicated widespread presence of filarial DNA in the mosquito population, suggesting persistent low-level transmission of LF on Tutuila and Aunu’u. [27]

For our study, mosquito data were summarized for each village for i) *Ae*. *polynesiensis*, and ii) other mosquito species (all species apart from *Ae*. *polynesiensis*), and iii) any mosquito species. Entomological data available by village included number of traps used; number of females and pools of each mosquito species; number of PCR-positive pools of each species; and estimated prevalence of PCR-positive *Ae*. *polynesiensis* (using PoolScreen software).

### Ethical considerations

This study used de-identified data from the two previously published studies described above [7,28]. The human study only included adults, and written informed consent was obtained from each participant. The American Samoa Institutional Review Board (IRB) provided approval for the use of the human serum bank for lymphatic filariasis research.

### Data analysis

In the MX study, some small adjacent villages were combined into groups of two to four villages for trapping and analyses, and human data were grouped accordingly to match the entomological data. Human data were not available for three of the villages included in the MX study. For this study, analyses were limited to the villages or village groups where both human data and MX data were available for 32 locations: 23 individual villages on Tutuila, 3 village groups (of two villages each) on Tutuila, the only village on Aunu’u, and all five villages on the Manu’a Islands. The 32 villages and village groups will be referred to as “villages” from here for ease of reference.

Chi-squared tests and logistic regression were used to identify associations between seropositive humans and PCR-positive mosquito pools, and answer the following operational questions:

- Is the presence of PCR-positive pools of *Ae*. *polynesiensis* in a village a useful indicator of a seropositive village? If so, how accurate are PCR-positive pools for predicting seropositive villages for Og4C3 Ag, Wb123 Ab, and Bm14 Ab?
- Is the presence of PCR-positive pools of *Ae*. *polynesiensis* a better indicator of seropositive villages than PCR-positive pools of other mosquito species, or PCR-positive pools of any mosquito species? In other words, do the time, effort, and expertise required to separate mosquitoes into species-specific pools improve the accuracy of the predictions?
- Is the estimated prevalence of PCR-positive *Ae*. *polynesiensis* (calculated by PoolScreen) a better indicator of the above measures? In other words, is it necessary to estimate prevalence using PoolScreen, or does the presence/absence of PCR-positive pools provide equally accurate predictions?

## Results

Serological results from 376 persons residing in 32 villages were included in the analyses. The average number of persons per village was 13.9 (range 2–73) for Tutuila and Aunu’u, and 14.0 (range 11–16) for the Manu’a Islands.

Table 1 provides a summary of the number of seropositive persons and village-level seroprevalence for each serological marker in humans, and the entomological data used in this study. Of the 32 villages included in this study, 11 (34.4%) had residents who were seropositive for Og4C3 Ag, 18 (56.3%) for Wb123 Ab, and 27 (84.4%) for Bm14 Ab. On Tutuila and Aunu’u, village-level seroprevalence ranged from 0% to 33.3% for Og4C3 Ag, 0% to 66.7% for Wb123 Ab, and 0% to 100% for Bm14 Ab. In the Manu’a Islands, no individuals were seropositive for Og4C3 Ag, and village-level seroprevalence ranged from 0% to 18.8% for Wb123 Ab, and 13.3% to 27.3% for Bm14 Ab. On Tutuila and Aunu’u, the MX study identified PCR-positive pools of *Ae*. *polynesiensis* in 15 (55.6%) of the 27 villages included in this study, of other mosquito species in 7 (25.9%) villages, and of mosquitoes of any species in 17 (63.0%) of the villages. In the five villages on the Manu’a Islands, no PCR-positive pools of mosquitoes were identified during the MX study.

**Table 1:** Summary of human and entomological data from the 32 villages included in this study.

Islands	Total number of study villages in island group	Number of villages with persons sero-positive for Og4C3 Ag (%)	Number of villages with persons sero-positive for Wb123 Ab (%)	Number of villages with persons sero-positive for Bm14 Ab (%)	Mean village-level sero-prevalence of Og4C3 Ag (range)	Mean village-level sero-prevalence of Wb123 Ab (range)	Mean village-level sero-prevalence of Bm14 Ab (range)	Mean number of mosquito pools per village (range)	Villages with ≥1 PCR-positive pool of *Ae*. *polynesiensis* N (%)	Villages with ≥1 PCR-positive pool of other mosquito species N (%)	Villages with ≥1 PCR-positive pool of any mosquito species N (%)	Mean point estimates of prevalence of PCR-positive *Ae*. polynesiensis* (range)
**Tutuila & Aunu’u**	27	11 (40.7%)	15 (55.6%)	22 (81.5%)	4.8% (0–33.3%)	9.3% (0–66.7%)	18.4% (0–100%)	80.5 (12–133)	15 (55.6%)	7 (25.9%)	17 (63.0%)	0.5% (0–2.8%)
**Manu’a**	5	0 (0%)	3 (60.0%)	5 (100%)	0% (N/A)	8.6% (0–18.8%)	21.4% (13.3–27.3%)	84.0 (44–119)	0 (0%)	0 (0%)	0 (0%)	0% (0%)
**Total**	32	11 (34.4%)	18 (56.3%)	27 (84.4%)	4.0%	9.2%	18.8%	81.1	15 (46.9%)	7 (21.9%)	17 (53.1%)	0.4% (0–2.8%)

* Calculated using PoolScreen software in molecular xenomonitoring study [28]

### Association between PCR-positive mosquito pools and seropositive villages

Associations between the presence of PCR-positive pools of mosquitoes and seropositive villages and are shown in Table 2, with analyses stratified for i) *Ae*. *polynesiensis* only, ii) for all other mosquito species and iii) for mosquitoes of any species. Chi-squared tests of association showed that PCR-positive pools of *Ae*. *polynesiensis* (p ≤ 0.001) and PCR-positive pools of any species (p = 0.002), but not pools of other species, were significantly associated with seropositive villages for Og4C3 Ag and Wb123 Ab, but not Bm14 Ab.

**Table 2:** Association between PCR-positive pools of mosquitoes and seropositive villages for Og4C3 Ag, Wb123 Ab, and Bm14 Ab.

Antigen/antibody status of villages^#^	Number of villages (% of total)	Villages with PCR-positive pools of *Ae*. *polynesiensis*		Villages with PCR-positive pools of other species		Villages with PCR-positive pools of any species	
		N (%)	*p* value*	N (%)	*p* value*	N (%)	*p* value*
Total villages	32 (100%)	**15 (100%)**		**7 (100%)**		**17 (100%)**	
Seropositive for Og4C3 Ag	11 (34.4%)	10 (66.7)	**<0.001**	4 (57.1)	0.151	10 (58.8)	**0.002**
Seronegative for Og4C3 Ag	21 (65.6%)	5 (33.3)		3 (42.9)		7 (41.2)	
Seropositive for Wb123 Ab	18 (56.3%)	13 (86.7)	**0.001**	6 (85.7)	0.075	14 (82.4)	**0.002**
Seronegative for Wb123 Ab	14 (48.8%)	2 (13.3)		1 (14.3)		3 (17.6)	
Seropositive for Bm14 Ab	27 (84.4%)	13 (86.7)	0.737	6 (85.7)	0.912	14 (82.4)	0.737
Seronegative for Bm14 Ab	5 (15.6%)	2 (13.3)		1 (14.3)		3 (17.6)	

^#^ A seropositive village is defined as a village with at least one seropositive person for the antigen or antibody. A seronegative village is defined as a village with no seropositive persons.

*Chi-squared tests comparison of villages with presence/absence of PCR-positive mosquito pools and presence/absence of seropositive persons. Statistically significant results highlighted in bold.

**Fig 1:**
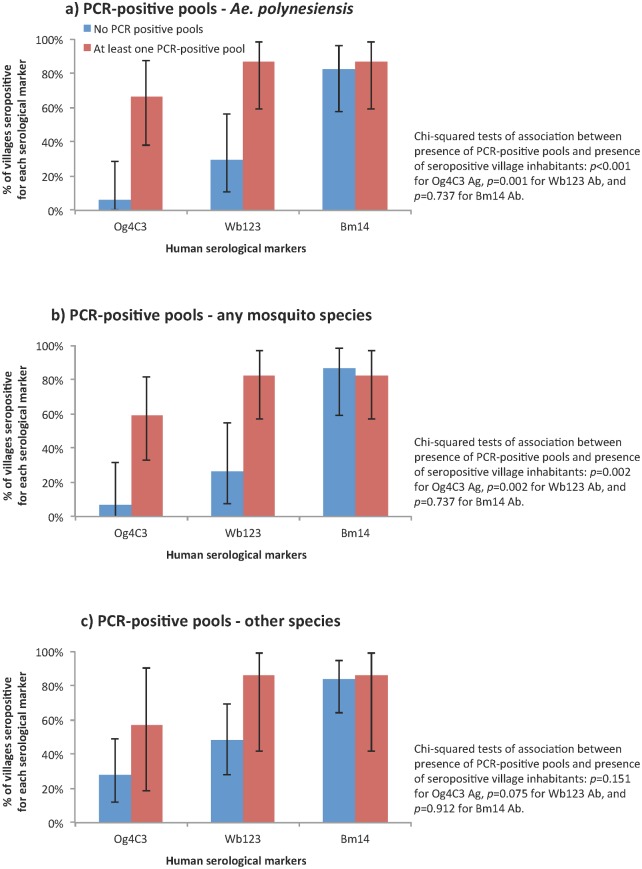
Probabilities of identifying seropositive villages for Og4C3 Ag, Wb123 Ab and Bm14 Ab based on the presence of PCR-positive pools of a) Ae. polynesiensis, b) any mosquito species, and c) other mosquito species.

Fig 1 shows that the presence of at least one PCR-positive pool of *Ae*. *polynesiensis* or of any species was associated with a significantly higher probability of identifying a village with inhabitants seropositive for Og4C3 Ag (*p* < 0.001 and *p* = 0.002) and Wb123 Ab (*p* = 0.001 and *p* = 0.002). In the 15 villages with at least one PCR-positive pool of *Ae*. *polynesiensis*, 10 (67%) were seropositive for Og4C3 Ag and 13 (87%) were seropositive for Wb123 Ab, compared to 6% and 29% of villages respectively, where PCR-positive pools were not identified. Similarly, in the 17 villages where at least one PCR-positive pool of any species were identified, 11 (59%) had inhabitants who were seropositive for Og4C3 Ag and 14 (82%) with persons seropositive for Wb123 Ab, compared to 7% and 27% of villages respectively, with no PCR-positive pools. The presence of PCR-positive pools was not significantly associated with seropositivity for Bm14 Ab. PCR-positive pools of other mosquito species were not significantly associated with seropositive villages for any of the serological markers.

### Predicting the location of seropositive villages by using the presence of PCR-positive pools of mosquitoes

Table 3 provides a summary of the accuracy of PCR-positive mosquito pools for predicting seropositive villages for each antigen and antibody. PCR-positive pools of *Ae*. *polynesiensis* provide a sensitivity of 90.9% and specificity of 76.2% for identifying villages with seropositive persons for Og4C3 Ag, with a high negative predictive value of 94.1% (i.e. absence of PCR-positive pools was a good indicator of the absence of seropositive persons). PCR-positive pools of any mosquito species provide the same sensitivity (90.9%) but a lower specificity (66.7%), and a negative predictive value of 93.9%.

**Table 3:** PCR-positive pools of mosquitoes as predictors of villages with inhabitants seropositive for Og4C3 Ag, Wb123 Ab, and Bm14 Ab.

	Any PCR-positive pools	Sensitivity	Specificity	Positive predictive value	Negative predictive value	Odds ratio* (95% CI)	*p* value*
**a) Seropositive villages for Og4C3 Ag**	*Ae*. *polynesiensis*	90.9%	76.2%	66.7%	94.1%	32.0 (3.2–315.3)	**0.003**
	Other mosquito species	36.4%	85.7%	57.1%	72.0%	3.43 (0.6–19.4)	0.163
	Any mosquito species	90.9%	66.7%	58.8%	93.3%	20.0 (2.1–189.2)	**0.009**
**b) Seropositive villages for Wb123 Ab**	*Ae*. *polynesiensis*	72.2%	85.7%	86.7%	70.6%	15.6 (2.5–96.1)	**0.003**
	Other mosquito species	33.3%	92.9%	85.7%	52.0%	6.5 (0.7–62.1)	0.104
	Any mosquito species	77.8%	78.6%	82.4%	73.3%	12.8 (2.4–69.7)	**0.003**
**c) Seropositive villages for Bm14 Ab**	*Ae*. *polynesiensis*	48.1%	60.0%	86.7%	17.6%	1.4 (0.2–9.7)	0.738
	Other mosquito species	22.2%	80.8%	85.7%	16.0%	1.1 (0.1–12.2)	0.912
	Any mosquito species	51.9%	40.0%	82.4%	13.3%	0.7 (0.1–5.0)	0.738

*Odds ratio of seropositive village if PCR-positive mosquitoes were identified (logistic regression), and associated *p* value (statistically significant results highlighted in bold).

For Wb123 Ab, PCR-positive pools of *Ae*. *polynesiensis* provide a sensitivity of 72.2% and specificity of 85.7%, while PCR-positive pools of any mosquito species provide a sensitivity of 77.8% and specificity of 78.6% for identifying seropositive villages. For Bm14 Ab, PCR-positive pools of *Ae*. *polynesiensis* and any species had poor sensitivities (48.1% and 51.9%) and specificities (60.0% and 40.0%) for predicting seropositive villages.

**Fig 2:**
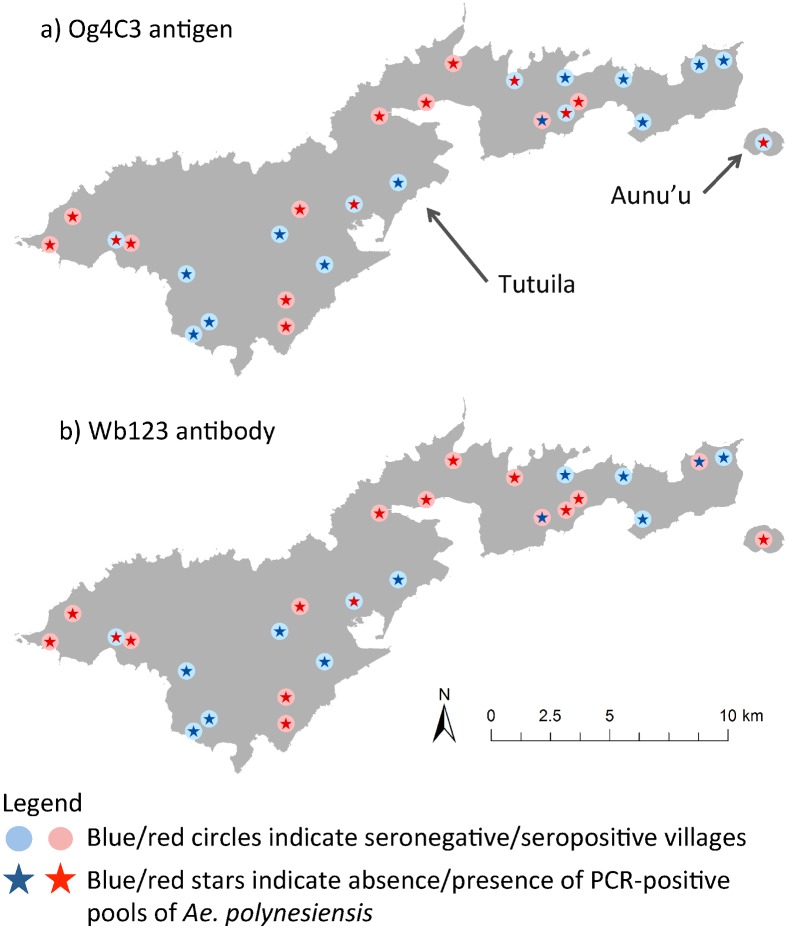
Associations between PCR-positive pools of Ae. polynesiensis and seropositive villages for Og4C3 Ag and Wb123 Ab on Tutuila and Aunu’u.

**Fig 3:**
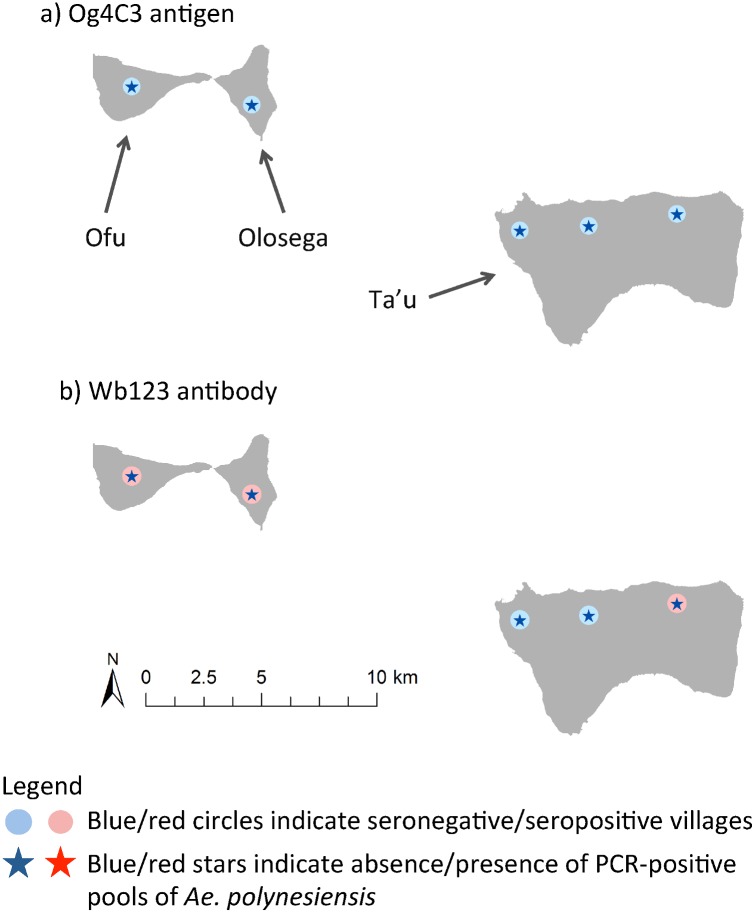
Association between PCR-positive pools of Ae. polynesiensis and seropositive villages for Og4C3 Ag and Wb123 Ab on the Manu’a Islands.

PCR-positive pools of *Ae*. *polynesiensis* or any mosquito species were statistically significant predictors of villages with residents seropositive for Og4C3 Ag (odds ratios of 32.0 and 20.0) and Wb123 Ab (odds ratios of 15.6 and 12.8), but not for Bm14 Ab. The correlation between PCR-positive pools of *Ae*. *polynesiensis* and seropositive villages for Og4C3 Ag and Wb123 Ab are shown for each village in Tutuila and Aunu’u in Fig 2, and the Manu’a Islands in Fig 3.

### Predicting the location of seropositive villages by using the estimated prevalence of PCR-positive *Ae*. *polynesiensis*

In the MX study, the estimated prevalence of PCR-positive *Ae*. *polynesiensis* ranged from 0% (95% CI 0–0.1%) to 2.8% (0.5–7.9%) on Tutuila and Aunu’u, and was 0% for all villages in the Manu’a islands. Table 4 shows that a higher estimated prevalence of PCR-positive *Ae*. *polynesiensis* (as a continuous variable) was associated with increased odds of a seropositive village for Og4C3 Ag and Wb123 Ab, but the findings were not statistically significant with this sample size and the level of precision inherent in PoolScreen predictions based on pooled mosquito samples.

**Table 4:** Association between estimated prevalence of PCR-positive *Ae*. *polynesiensis* (using PoolScreen) and seropositive villages for Og4C3 Ag, Wb123 Ab, and Bm14 Ab.

Seropositive villages for	Odds ratio*	95% CI	*p* value
**Og4C3**	1.76	0.51–6.03	0.367
**Wb123**	1.85	0.46–7.34	0.384
**Bm14**	0.42	0.11–1.64	0.211

* Odds ratio on logistic regression, per 1% increase in estimated prevalence of PCR-positive *Ae*. *polynesiensis* (calculated using PoolScreen).

## Discussion

Our results show that MX is a potentially useful tool for post-MDA surveillance of lymphatic filariasis in American Samoa. The presence of PCR-positive pools of *Ae*. *polynesiensis* was found to be a good predictor of villages with persons seropositive for Og4C3 Ag and Wb123 Ab, but not Bm14 Ab. Bm14 Ab can persist for many years or decades after initial infection but does not necessarily persist for life, and antibody levels can also decline or be cleared after MDA [36,37]. Wb123 Ab can also persist for many years after initial infection, and declines after MDA [38]. Currently, there is insufficient data on the relative rates of antibody decay or clearance, but it is thought that Wb123 Ab responses decay more rapidly than Bm14 Ab. Biologically, Wb123 Ab responses might increase earlier because they are against a larval antigen and therefore also more likely to be associated with mosquito exposure. In our study, the lack of association between PCR-positive pools of mosquitoes and Bm14 Ab was therefore not unexpected, but the association with Wb123 Ab could be related to earlier appearance or faster disappearance of Wb123 Ab than Bm14 Ab during and after active infections, respectively [39–41].

In American Samoa, where ~75% of mosquitoes collected in the entomology study (using BG Sentinel traps) were *Ae*. *polynesiensis*, the presence of PCR-positive pools of either *Ae*. *polynesiensis* alone or any mosquito species provided similar predictive accuracy of identifying villages with residents seropositive for Og4C3 Ag or Wb123 Ab. Our study shows that in this setting, separation of mosquito species for MX did not improve the predictive accuracy for identifying villages with seropositive inhabitants. However, it is important to point out that our results would have been different if we had used traps with a different level of selectivity for *Ae*. *polynesisensis*. MX studies in locations with other vector species and employing different traps may require sorting of mosquito species to achieve optimal results.

The presence of PCR-positive pools of *Ae*. *polynesiensis* or mosquitoes of any species had high sensitivity and high negative predictive value (both >90%) for correctly identifying villages with antigen-positive persons. These are both important for post-MDA surveillance because tests should have a high probability of identifying residual foci of transmission (when prevalence is very low) and low probability of missing these foci. In this study, the estimated prevalence of PCR-positive *Ae*. *polynesiensis* (using PoolScreen) was no more useful than the presence/absence of PCR-positive pools, but the sampling design (small number of persons in some villages) might have limited the ability to detect significant associations.

Previous studies in three sentinel villages in American Samoa showed that MX could be a useful tool in post-MDA surveillance [11,18]; our larger study of 32 villages corroborates those conclusions. Our findings also suggest that in American Samoa, it is appropriate to conduct post-MDA surveillance at the village level. This is biologically plausible considering that the main vector, *Ae*. *polynesiensis*, has a relatively short flight range of about 100 metres [17], and village residents are generally quite mobile within their own village, e.g. visiting homes of family and friends, sharing outdoor spaces, attending school and church, and shopping at local stores. Further interventions (e.g. further targeted MDA or a test and treat approach) could also be conducted at the village or even sub-village level.

The results should be considered in light of the study’s limitations. The study was based on serological data from humans; microfilaria results were not available because the study was conducted using a pre-existing serum bank. Human serological data and entomological data were sourced from previously published studies, and there was a time lag of approximately nine months between the human and entomology studies. Sampling of the human seroprevalence study was designed to maximise spatial dispersion for the purposes of predictive risk mapping for the original leptospirosis study [7], resulting in small numbers of subjects in some villages and wide confidence intervals for the village-level LF seroprevalence estimates. The serum bank only included samples from adults (aged ≥ 18 years); a study that focused on or only included children might produce different results regarding the usefulness of serological markers, e.g. there could be significant associations between PCR-positive mosquitoes and Bm14 Ab in children. *Ae*. *polynesiensis*, the primary vector in American Samoa, is a day-biting mosquito; our human data were summarised by village of residence, and it is possible that PCR-positive mosquitoes acquired infections from residents of other villages who visited during day time.

Despite the study’s limitations, we were able to identify statistically significant associations between MX data and human seroprevalence data at the village level. Further studies specifically designed to assess the usefulness of MX in the post-MDA setting might produce results with even stronger associations. With higher resolution data, it is also potentially possible to determine thresholds for the prevalence of PCR-positive *Ae*. *polynesiensis* at which further interventions (e.g. repeating MDA or more intensive surveillance) are recommended. In American Samoa, LF transmission is dominated by *Ae*. *polynesiensis*; studies in other countries with a different mix of vector mosquitoes will be needed to determine whether separation of mosquitoes by species is necessary for MX.

This exploratory study provides promising evidence to support the potential usefulness of MX in post-MDA surveillance in an *Aedes* transmission area in a Pacific Island setting to predict sub-national areas where LF transmission may still be occurring. Although American Samoa has successfully completed MDA and passed two TAS of 6–7 year old children, there is evidence of ongoing low-level transmission of LF. Our findings demonstrated that in this setting, MX was useful for localising residual areas of focal transmission and could potentially be used to inform the need for additional elimination activities. Our study also highlights that assessment of antigen prevalence in adults in post-MDA surveillance could complement TAS and provide valuable information for informing programmatic decisions in the endgame.

## Supporting Information

S1 Appendix Village-level human serological data for Og4C3, Wb123 and Bm14. (XLSX)

S1 Checklist STROBE statement for cross-sectional studies. (DOC)

## References

[1] Ramaiah KD, Ottesen EA. Progress and Impact of 13 Years of the Global Programme to Eliminate Lymphatic Filariasis on Reducing the Burden of Filarial Disease. PLoS Negl Trop Dis. 2014;8: e3319 10.1371/journal.pntd.0003319 25412180 PMC4239120

[2] Ichimori K, Crump A. Pacific Collaboration to Eliminate Lymphatic Filariasis. Trends in Parasitology. 2005;21: 441–444. 10.1016/j.pt.2005.08.010 16099721 PMC7185803

[3] World Health Organization. Monitoring and Epidemiological Assessment of Mass Drug Administration in the Global Programme to Eliminate Lymphatic Filariasis: A Manual for National Elimination Programmes. http://apps.who.int/iris/bitstream/10665/44580/1/9789241501484_eng.pdf

[4] Weil GJ, Curtis KC, Fakoli L, Fischer K, Gankpala L, et al Laboratory and Field Evaluation of a New Rapid Test for Detecting Wuchereria Bancrofti Antigen in Human Blood. Am J Trop Med Hyg. 2013;89: 11–15. 10.4269/ajtmh.13-0089 23690552 PMC3748464

[5] Gounoue-Kamkumo R, Nana-Djeunga HC, Bopda J, Akame J, Tarini A, et al Loss of Sensitivity of Immunochromatographic Test (Ict) for Lymphatic Filariasis Diagnosis in Low Prevalence Settings: Consequence in the Monitoring and Evaluation Procedures. BMC Infect Dis. 2015;15: 579 10.1186/s12879-015-1317-x 26700472 PMC4690254

[6] Njenga SM, Wamae CN, Njomo DW, Mwandawiro CS, Molyneux DH. Impact of Two Rounds of Mass Treatment with Diethylcarbamazine Plus Albendazole on Wuchereria Bancrofti Infection and the Sensitivity of Immunochromatographic Test in Malindi, Kenya. Trans R Soc Trop Med Hyg. 2008;102: 1017–1024. 10.1016/j.trstmh.2008.04.039 18550135

[7] Lau CL, Won KY, Becker L, Soares Magalhaes RJ, Fuimaono S, et al Seroprevalence and Spatial Epidemiology of Lymphatic Filariasis in American Samoa after Successful Mass Drug Administration. PLoS Negl Trop Dis. 2014;8: e3297 10.1371/journal.pntd.0003297 25393716 PMC4230933

[8] Rao RU, Nagodavithana KC, Samarasekera SD, Wijegunawardana AD, Premakumara WD, et al A Comprehensive Assessment of Lymphatic Filariasis in Sri Lanka Six Years after Cessation of Mass Drug Administration. PLoS Negl Trop Dis. 2014;8: e3281 10.1371/journal.pntd.0003281 25393404 PMC4230885

[9] World Health Organization. Lymphatic Filariasis—Research. 2016. http://www.who.int/lymphatic_filariasis/research/en/

[10] Bockarie MJ. Molecular Xenomonitoring of Lymphatic Filariasis. Am J Trop Med Hyg. 2007;77: 591–592. 17978054

[11] Mladonicky JM, King JD, Liang JL, Chambers E, Pa'au M, et al Assessing Transmission of Lymphatic Filariasis Using Parasitologic, Serologic, and Entomologic Tools after Mass Drug Administration in American Samoa. Am J Trop Med Hyg. 2009;80: 769–773. 19407122

[12] Farid HA, Morsy ZS, Helmy H, Ramzy RM, El Setouhy M, et al A Critical Appraisal of Molecular Xenomonitoring as a Tool for Assessing Progress toward Elimination of Lymphatic Filariasis. Am J Trop Med Hyg. 2007;77: 593–600. 17978055 PMC2196407

[13] Pedersen EM, Stolk WA, Laney SJ, Michael E. The Role of Monitoring Mosquito Infection in the Global Programme to Eliminate Lymphatic Filariasis. Trends Parasitol. 2009;25: 319–327. 10.1016/j.pt.2009.03.013 19559649

[14] Williams SA, Laney SJ, Bierwert LA, Saunders LJ, Boakye DA, et al Development and Standardization of a Rapid, Pcr-Based Method for the Detection of Wuchereria Bancrofti in Mosquitoes, for Xenomonitoring the Human Prevalence of Bancroftian Filariasis. Ann Trop Med Parasitol. 2002;96 Suppl 2: S41–46. 10.1179/000349802125002356 12625916

[15] Goodman DS, Orelus JN, Roberts JM, Lammie PJ, Streit TG. Pcr and Mosquito Dissection as Tools to Monitor Filarial Infection Levels Following Mass Treatment. Filaria J. 2003;2: 11 10.1186/1475-2883-2-11 12890288 PMC169178

[16] Plichart C, Sechan Y, Davies N, Legrand AM. Pcr and Dissection as Tools to Monitor Filarial Infection of Aedes Polynesiensis Mosquitoes in French Polynesia. Filaria J. 2006;5: 2 10.1186/1475-2883-5-2 16504131 PMC1403774

[17] Jachowski LA Jr. Filariasis in American Samoa. V. Bionomics of the Principal Vector, Aedes Polynesiensis Marks. American journal of hygiene. 1954;60: 186–203. 13197370

[18] Chambers EW, McClintock SK, Avery MF, King JD, Bradley MH, et al Xenomonitoring of Wuchereria Bancrofti and Dirofilaria Immitis Infections in Mosquitoes from American Samoa: Trapping Considerations and a Comparison of Polymerase Chain Reaction Assays with Dissection. The American journal of tropical medicine and hygiene. 2009;80: 774–781. 19407123

[19] Nicolas L, Luquiaud P, Lardeux F, Mercer DR. A Polymerase Chain Reaction Assay to Determine Infection of Aedes Polynesiensis by Wuchereria Bancrofti. Trans R Soc Trop Med Hyg. 1996;90: 136–139. 8761572 10.1016/s0035-9203(96)90113-3

[20] Fischer P, Erickson SM, Fischer K, Fuchs JF, Rao RU, et al Persistence of Brugia Malayi DNA in Vector and Non-Vector Mosquitoes: Implications for Xenomonitoring and Transmission Monitoring of Lymphatic Filariasis. The American journal of tropical medicine and hygiene. 2007;76: 502–507. 17360875 PMC2196403

[21] Farid HA, Hammad RE, Hassan MM, Morsy ZS, Kamal IH, et al Detection of Wuchereria Bancrofti in Mosquitoes by the Polymerase Chain Reaction: A Potentially Useful Tool for Large-Scale Control Programmes. Trans R Soc Trop Med Hyg. 2001;95: 29–32. 11280059 10.1016/s0035-9203(01)90322-0

[22] de Souza DK, Ansumana R, Sessay S, Conteh A, Koudou B, et al The Impact of Residual Infections on Anopheles-Transmitted Wuchereria Bancrofti after Multiple Rounds of Mass Drug Administration. Parasit Vectors. 2015;8: 488 10.1186/s13071-015-1091-z 26399968 PMC4581406

[23] Owusu IO, de Souza DK, Anto F, Wilson MD, Boakye DA, et al Evaluation of Human and Mosquito Based Diagnostic Tools for Defining Endpoints for Elimination of Anopheles Transmitted Lymphatic Filariasis in Ghana. Trans R Soc Trop Med Hyg. 2015;109: 628–635. 10.1093/trstmh/trv070 26385935

[24] Weil GJ, Ramzy RM. Diagnostic Tools for Filariasis Elimination Programs. Trends Parasitol. 2007;23: 78–82. 10.1016/j.pt.2006.12.001 17174604

[25] Michael E, Malecela-Lazaro MN, Kabali C, Snow LC, Kazura JW. Mathematical Models and Lymphatic Filariasis Control: Endpoints and Optimal Interventions. Trends Parasitol. 2006;22: 226–233. 10.1016/j.pt.2006.03.005 16564745

[26] World Health Organization. The Role of Polymerase Chain Reaction Techniques for Assessing Lymphatic Filariasis Transmission. 2006. http://apps.who.int/iris/bitstream/10665/70255/1/WHO_HTM_NTD_PCT_2009.1_eng.pdf

[27] WHO Western Pacific Region DoPTS. Pacific Programme to Eliminate Lymphatic Filariasis. 2013. http://www.wpro.who.int/southpacific/pacelf/en/

[28] Schmaedick MA, Koppel AL, Pilotte N, Torres M, Williams SA, et al Molecular Xenomonitoring Using Mosquitoes to Map Lymphatic Filariasis after Mass Drug Administration in American Samoa. PLoS Negl Trop Dis. 2014;8: e3087 10.1371/journal.pntd.0003087 25122037 PMC4133231

[29] American Samoa Department of Commerce. 2012 Statistical Yearbook. 2012. http://americansamoa.gov/index.php/2012-04-25-19-44-32/2012-04-25-19-52-04/departments/commerce

[30] Ramalingam S, Belkin JN. Vectors of Sub-Periodic Bancroftian Filariasis in the Samoa-Tonga Area. Nature. 1964;201: 105–106. 14085555 10.1038/201105b0

[31] Ramalingam S. The Epidemiology of Filarial Transmission in Samoa and Tonga. Ann Trop Med Parasitol. 1968;62: 305–324. 5751950 10.1080/00034983.1968.11686565

[32] Samarawickrema WA, Sone F, Cummings RF. Natural Infections of Wuchereria Bancrofti in Aedes (Stegomyia) Polynesiensis and Aedes (Finlaya) Samoanus in Samoa. Trans R Soc Trop Med Hyg. 1987;81: 124–128. 3328327 10.1016/0035-9203(87)90303-8

[33] Lau CL, Dobson AJ, Smythe LD, Fearnley EJ, Skelly C, et al Leptospirosis in American Samoa 2010: Epidemiology, Environmental Drivers, and the Management of Emergence. Am J Trop Med Hyg. 2012;86: 309–319. 10.4269/ajtmh.2012.11-0398 22302868 PMC3269286

[34] Lau CL, Clements AC, Skelly C, Dobson AJ, Smythe LD, et al Leptospirosis in American Samoa—Estimating and Mapping Risk Using Environmental Data. PLoS Neg Trop Dis. 2012;6: e1669. 10.1371/journal.pntd.0001669 PMC3362644 22666516

[35] Rao RU, Atkinson LJ, Ramzy RM, Helmy H, Farid HA, et al A Real-Time Pcr-Based Assay for Detection of Wuchereria Bancrofti DNA in Blood and Mosquitoes. Am J Trop Med Hyg. 2006;74: 826–832. 16687688 PMC2196401

[36] Helmy H, Weil GJ, Ellethy AS, Ahmed ES, Setouhy ME, et al Bancroftian Filariasis: Effect of Repeated Treatment with Diethylcarbamazine and Albendazole on Microfilaraemia, Antigenaemia and Antifilarial Antibodies. Trans R Soc Trop Med Hyg. 2006;100: 656–662. 10.1016/j.trstmh.2005.08.015 16414095

[37] Weil GJ, Kastens W, Susapu M, Laney SJ, Williams SA, et al The Impact of Repeated Rounds of Mass Drug Administration with Diethylcarbamazine Plus Albendazole on Bancroftian Filariasis in Papua New Guinea. PLoS Negl Trop Dis. 2008;2: e344 10.1371/journal.pntd.0000344 19065257 PMC2586652

[38] Steel C, Kubofcik J, Ottesen EA, Nutman TB. Antibody to the Filarial Antigen Wb123 Reflects Reduced Transmission and Decreased Exposure in Children Born Following Single Mass Drug Administration (Mda). PLoS Negl Trop Dis. 2012;6: e1940 10.1371/journal.pntd.0001940 23236533 PMC3516579

[39] Steel C, Golden A, Kubofcik J, LaRue N, de Los Santos T, et al Rapid Wuchereria Bancrofti-Specific Antigen Wb123-Based Igg4 Immunoassays as Tools for Surveillance Following Mass Drug Administration Programs on Lymphatic Filariasis. Clin Vaccine Immunol. 2013;20: 1155–1161. 10.1128/CVI.00252-13 23740923 PMC3754496

[40] Kubofcik J, Fink DL, Nutman TB. Identification of Wb123 as an Early and Specific Marker of Wuchereria Bancrofti Infection. PLoS Negl Trop Dis. 2012;6: e1930 10.1371/journal.pntd.0001930 23236529 PMC3516582

[41] Hamlin KL, Moss DM, Priest JW, Roberts J, Kubofcik J, et al Longitudinal Monitoring of the Development of Antifilarial Antibodies and Acquisition of Wuchereria Bancrofti in a Highly Endemic Area of Haiti. PLoS Negl Trop Dis. 2012;6: e1941 10.1371/journal.pntd.0001941 23236534 PMC3516578

